# Sherborn’s *Index Animalium*: New names, systematic errors and availability of names in the light of modern nomenclature

**DOI:** 10.3897/zookeys.550.10041

**Published:** 2016-01-07

**Authors:** Francisco Welter-Schultes, Angela Görlich, Alexandra Lutze

**Affiliations:** 1 Zoologisches Institut der Universität, Berliner Strasse 28, D-37073 Göttingen, Germany

**Keywords:** Sherborn

## Abstract

This study is aimed to shed light on the reliability of Sherborn’s *Index Animalium* in terms of modern usage. The AnimalBase project spent several years’ worth of teamwork dedicated to extracting new names from original sources in the period ranging from 1757 to the mid-1790s. This allowed us to closely analyse Sherborn’s work and verify the completeness and correctness of his record. We found the reliability of Sherborn’s resource generally very high, but in some special situations the reliability was reduced due to systematic errors or incompleteness in source material. *Index Animalium* is commonly used by taxonomists today who rely strongly on Sherborn’s record; our study is directed most pointedly at those users. We recommend paying special attention to the situations where we found that Sherborn’s data should be read with caution.

In addition to some categories of systematic errors and mistakes that were Sherborn’s own responsibility, readers should also take into account that nomenclatural rules have been changed or refined in the past 100 years, and that Sherborn’s resource could eventually present outdated information. One of our main conclusions is that error rates in nomenclatoral compilations tend to be lower if one single and highly experienced person such as Sherborn carries out the work, than if a team is trying to do the task. Based on our experience with extracting names from original sources we came to the conclusion that error rates in such a manual work on names in a list are difficult to reduce below 2–4%. We suggest this is a natural limit and a point of diminishing returns for projects of this nature.

## Introduction

Appreciation of Sherborn’s tremendous work grows when we understand the extent to which Sherborn’s index data can be used for nomenclatural and taxonomic purposes today. However, some of the names listed in the *Index Animalium* should be used with caution. *Index Animalium* remains a core source of nomenclatural bibliographic information, but is not without errors.

In the AnimalBase project we manually checked original sources for approximately 40,000 names that were new between 1757 and 1795 and compared our results with those in Sherborn’s *Index Animalium*. For each examined work we extracted all new names under the present-day nomenclatural rules (4^th^ edition of the ICZN Code), and compared our results with Sherborn’s list extracted from the same work.

### Total numbers of names

It is crucial to know how to read the *Index Animalium*, as not all names were marked as new. Sherborn listed 420,000 names referenced between 1757 and 1850. 70% of these (300,000) were listed as new names from their original publications, however 30% of these names were not new.

**Figure 1.**
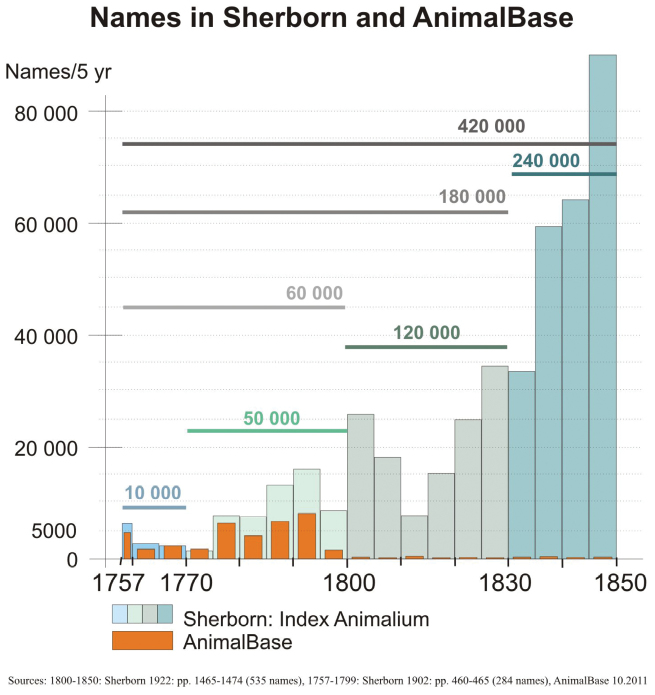
Number of names. Total number of taxonomic names listed in Sherborn’s *Index Animalium* and in AnimalBase (2011) plotted in 5-year intervals. It must be taken into account that Sherborn’s numbers included a proportion of 30% of names that were not new, while in AnimalBase this proportion was much lower (less than 5 %).

Taxonomic productivity, as indicated by number of new taxa described (Fig. 1), decreased temporarily around 1810 and increased enormously after 1835. After 1850 the rate did not continue to increase, but stayed at the same high level until around 1910 when it fell to levels of the 1820s. In the AnimalBase project we checked new names published between 1757 (the starting point for Code-regulated zoological nomenclature, ICZN Articles 3.1 and 3.2) and the mid-1790s, which was the historical limit for AnimalBase work for funding reasons. The numbers of listed names in this period listed 10-30% more names than found by AnimalBase, mainly because Sherborn included more *nomina nuda* (unavailable new names mentioned without description) than AnimalBase, and in addition many subsequent uses of previously established names.

### Understanding Sherborn’s style

Sherborn changed his standards various times in the course of his work. This sometimes gives the impression that he had a team and difficulties in reaching a common standard, however we know that he worked completely on his own (Evenhuis 2016, Shindler 2016). Initially he seemed to have recorded all subsequent uses of names, but this was given up (this was because he started with Linné (1766) and did not remove the entries when he decided to add those of the 1758 work). For a slightly longer period he recorded when specific names were placed in different genera. We have not been able to explain why *nomina nuda* or varieties were occasionally included. At later stages he only recorded new names and nothing more. When Sherborn was extracting names established in the 1790s he finally seemed to have reached a more stable standard.

We aim to shed light on Sherborn’s errors and their sources, as this will help taxonomists who use Sherborn’s data today to use it with greater reliability. With an understanding of systematic error sources, users can know when Sherborn’s record is at its most reliable, and when to proceed with some caution. We found that Sherborn’s data were consistent with our own finds at an average rate of 80–90%. The degree of reliability of Sherborn’s data differed by work and by animal group and depended on factors that we discuss below.

## Bibliographic methods and materials

How did Sherborn mark a name that was classified as an available new name, what was a subsequent use, a misspelling or a *nomen nudum*? Not all names in the *Index Animalium* were listed as new. We determined the rate of new names at roughly 70% of the listed names. 28–30% of the listed names were marked as subsequent uses and 2.5% as *nomina nuda*. Here are some examples of how to read Sherborn’s *Index Animalium* (our own comments below, after a hyphen):

aenea Nitidula, J. C. Fabricius, Syst. Ent. 1775, 78.

- New name, available.

carocolla Helix, Linnaeus, Syst Nat., ed. 10, 1758, 769 ; ed. 12, 1767, 1243.

- New name, available, with one subsequent use recorded for 1767.

haliaëtos Falco, J. F. Gmelin in Linn. Syst. Nat., ed. 13, I. 1788, 263 ; varr. arundinaceus, carolinensis, cayenensis.

- No new name *F. haliaetos*, but three new names of varieties (Sherborn regarded these names as unavailable; today they are regarded as available because of ICZN Art. 45.6.4)

aegyptius Lygaeus (L.), J. C. Fabricius, Ent. Syst. IV. 1794, 155.—Cimex.

- No new name, only a subsequent use of *Cimex aegyptius* Linnæus, 1758.

abbreviator Cryptus, J. C. Fabricius, Syst. Piezat. 1804, 84.—Ichneumon, 1798.

- No new name, only a subsequent use of *Ichneumon abbreviator* Fabricius, 1798.

aterrima Megilla (Panz.), J. C. Fabricius, Syst. Piezat. 1804, 331.—Apis, 1798.

- No new name, only a subsequent use of *Apis aterrima* Panzer, 1798.

abnormis Terebratula, M. Tuomey, Rep. Geol. S. Carolina, 1848, 209 [n. n.].

- No new name, not made available (nomen nudum).

carolina Manduca, J. Huebner, Exot. Schmett., Tab. Manduca [n. et f.].

- Name without description but with figure (nomen et figura) (Sherborn regarded this name as unavailable; today the name is regarded as available because of ICZN Art. 12.2.7).

### Differences in nomenclature between Sherborn’s times and today

Nomenclatural rules applied 100 years ago were different than those in force today. Sherborn began his work in the 1890s. The rules of zoological nomenclature were internationally fixed in 1905 (Blanchard et al. 1905), and not always in Sherborn’s sense. This generated errors for which Sherborn was not responsible as the criteria for availability of names, corrections of incorrect Latin, authorships for names, unavailability of non-binominal works had changed underneath him. In the following century the rules were continuously modified and refined. With every new edition of the ICZN Code the number of available names has changed again, despite all efforts to keep the status of those names stable which were made available under previous rules. Thousands of animal names still suffer from unclear regulations and different interpretations of ambiguous rules in the Code.

We looked closely at each name in an original copy of a published work and did not rely on Sherborn’s list. For example, Sherborn regarded names as available that had no description but listed only a host plant, which would be unavailable today. In contrast, he regarded names as unavailable that had no description but had a bibliographic reference to a description, but these would be regarded as available today. Sherborn usually did not list names of fossils or names of varieties, however today these are regarded as available names, in the technical sense of the Code.

Names for varieties were not usually listed as new by Sherborn. Names for varieties were mentioned from very early dates, for example those mentioned in Linné’s publications. Later (sometime between 1780 and 1800) Sherborn discontinued including variety names. This shows that over time Sherborn modified his own standards. As today these variety names are regarded as available, the consequences of Sherborn’s decision to exclude them created a systematic hole in the *Index Animalium*, with those names missing or listed from subsequent sources with incorrect dates.

### Names with host plants

Names that were only mentioned with a host plant, but without description, were generally listed as new by Sherborn who regarded such names as available. Today a new name that was published only with a host plant and not with a description, figure or indication, is regarded as unavailable (ICZN Code Art. 12.3).

### Names with figures

Names that were mentioned without description and that were only equipped with a figure were usually not listed as new by Sherborn as he did not regard them as available. Today a new name which was only published with a figure in the original source, is regarded as available (ICZN Art. 12.2.7).

### Names with references

New names that were mentioned without descriptions, but with bibliographical references to descriptions, were usually not listed as new by Sherborn as he regarded such names as unavailable. Today a new name published with a bibliographical reference in the original source is regarded as available (ICZN Art. 12.2.1).

### Non-binominal works

Names that were mentioned in important non-binominal or not consistently binominal works were inconsistently listed as new and available by Sherborn. Today a new name established in a non-binominal work is regarded as unavailable (ICZN Art. 11.4). The ICZN Code however does still not contain a precise definition of what exactly is a binominal name, so still today some works are under debate from the point of view of binominality. By attributing the new names to those works, Sherborn missed recording the first sources in binominal works where those names were later actually made available. Some examples for such non-binominal works are Geoffroy (1762), Hasselquist (1762), Gronovius (1763, 1781), Tunstall (1771), Zimmermann (1777), Meuschen (1778), and Martyn (1784).

### Correct spellings

Incorrect Latin was often corrected by Sherborn, who did not always copy the orthography exactly as used in the original source. This applied to some cases where Sherborn regarded the Latin names as incorrectly spelled. Today incorrect Latin is not corrected (ICZN Art. 32.5). Sherborn corrected incorrect Latin in only relatively few cases, but it makes *Index Animalium* unreliable to some degree. In questionable cases (if a name sounds unusual or has an unusual spelling) it is always necessary to consult the original sources. For example, *compunctus* was corrected to *compunctor*. The rate of such debatable names among all names is probably less than 1%, but these few names accumulate significantly among the names a taxonomist will consult when having been confronted with a spelling problem in the literature.

### Authorship

At the beginning of Sherborn’s work there were no universally agreed rules for nomenclature, and conventions for authorship of taxonomic names deviated substantially from today’s rules. Sherborn often attributed names to people to which names were attributed in original sources, but who could have been people who had not contributed to the descriptions. These people are usually called authors of manuscript names. Many manuscript names of molluscs were created by shell dealers (for example, Ziegler or Parress) who labelled specimens with self-invented names without having published anything. After the adoption of the first global rules of zoological nomenclature in 1905 it was determined that the authorship must be attributed to the scientist who published the first description (Blanchard et al. 1905). This basic rule has been maintained in all subsequent Codes (ICZN Art. 50). From 1905 onwards it has been globally accepted that the names should be attributed to the first scientists who have published a description, which in the mollusc examples were often E. A. Rossmässler or L. Pfeiffer.

### Error sources: understanding circumstances under which new names were systematically missed

Sherborn’s individual (i.e., non-systematic) error rate was remarkably low. His usual rate of overlooked names was 1–2%, and in carefully compiled works, it was sometimes even less. This was a very low rate for any human endeavour, let alone one of such monumental scale requiring detailed work over many decades. It is all the more impressive when we realise that today we have comparable failure rates, despite having many computer tools and in some cases teams of people to help with these issues.

The proportion of overlooked names from each source work depended on its style. We observed up to 40% overlooked names in chaotically arranged works such as Forskål (1775), Hartmann (1821) or Swainson (1840). We use the expression “chaotic work” for publications in which it is very difficult to detect a systematic arrangement of species accounts, different names for the same species were used in the same work, species were classified in different genera in the same work without visible preference, new names were not indicated as such and it was hardly visible if a name was new or not, and no systematic or alphabetic index was present.

However, even in some well-organised works Sherborn overlooked 2–5% of the new names for no apparent systematic reason. We were surprised to observe this in the extracted names of some of Fabricius’s insect works which all had a clear style. Our own error rates ranged at the same levels or higher. Sometimes names in *Index Animalium* were listed twice, with both correct and incorrect information.

There were however various systematic error sources that produced more damage to the reliability of the record.

### Languages

**Figure 2.**
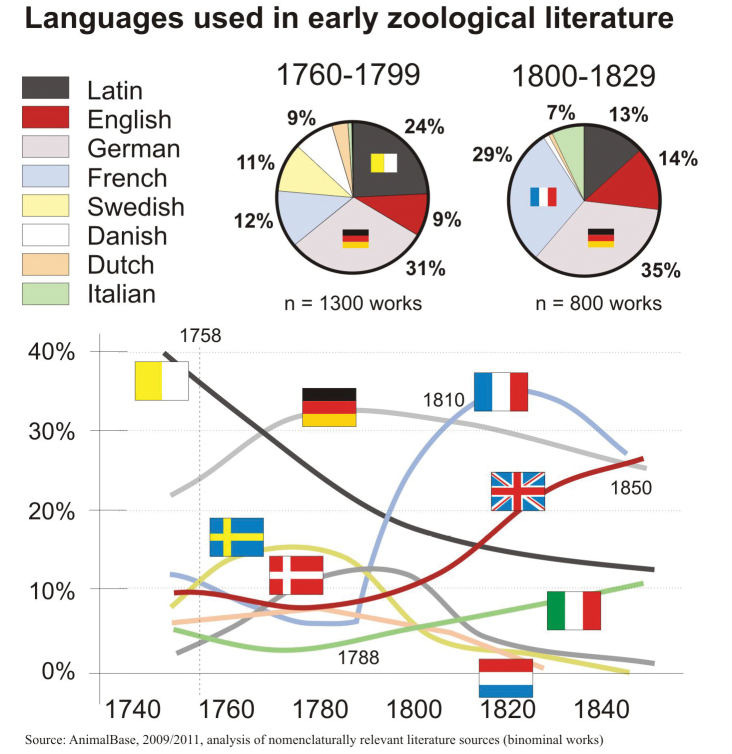
Languages used in early zoological literature. Language analysis of 2100 arbitrarily selected binominal zoological works published between 1758 and 1850.

Publications in languages that Sherborn had not mastered were a visible and significant problem. Sherborn was certainly well-versed in Latin, but had obvious difficulties in understanding all other languages except English. The problem was that only a fraction of the relevant works he had to consult was written in English or Latin (Fig. 2).

In the late 1700s German and Latin were the most commonly used languages for scientific publications. Even at that time, Latin was in continuous decline. Between 1760 and 1790 English was less frequently used than Swedish. Danish and Dutch were also important in this epoch. The importance of French in binominal zoological literature increased dramatically after Buffon’s death in 1788. Italian gained importance after 1820. This means that, at a minimum, Sherborn understood only one third of the published texts in the works from which he had to extract new names. Recognising names of new taxa was usually no problem, but understanding if a name was new or only a subsequent use of a previously established name (often transferred to a different genus) usually required understanding the context of descriptions.

In our survey of Sherborn’s error rates we were able to see that if images or Latin diagnoses were absent and texts were only in French, German, Dutch, Danish or Swedish, Sherborn often failed to draw the appropriate conclusions and consequently erroneously classified many names as new.

### Inaccessible works

Sherborn had unparalleled access to literature in London, but despite all efforts to get all published zoological literature, Sherborn did not have all relevant works at his disposal. Names established in missing works were not included in the *Index Animalium* and this often had tremendous impact: It distorted Sherborn’s work to a visible extent.

A striking example in the molluscan record is Férussac and Deshayes (1819–1851): *Histoire naturelle des mollusques*. This was an important basic work for molluscs in which 1% of the currently used names for species in Europe were established (and 5% of the names published before 1840). Sherborn did not have access to this work, so he missed many important names or listed them incorrectly as new from subsequent works. In combination with varieties having been ignored in most works, and careless research in difficult works like Hartmann (1821), this had the effect that today the *Index Animalium* is not widely considered as a reliable source for molluscs.

### Difficult works: Careless research as a strategy to save time

**Figure 3.**
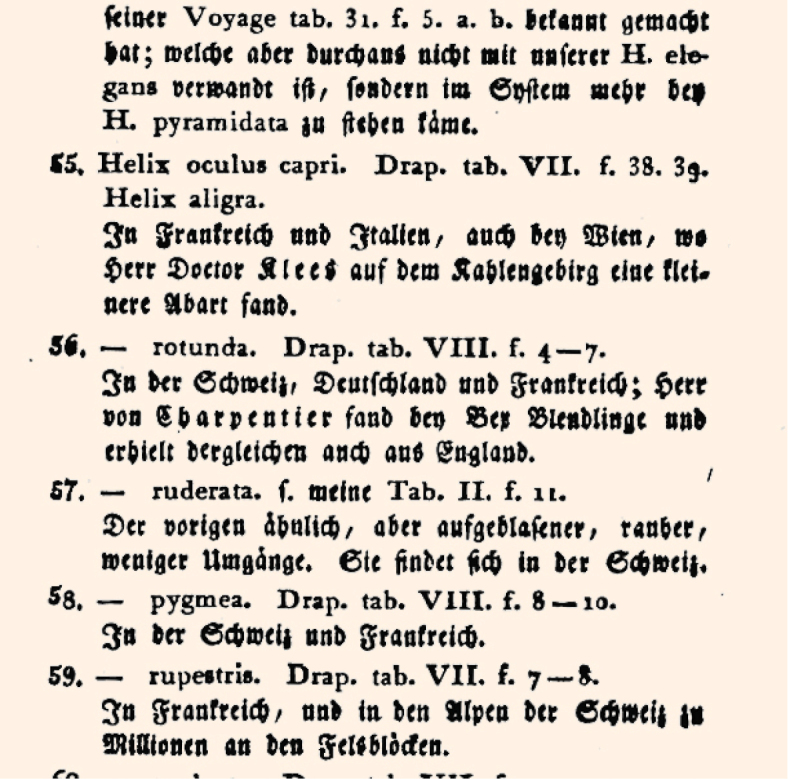
Example of a new name established in a chaotically arranged early zoological work. Detail of Hartmann (1821: p. 231), with the original description of *Helix ruderata* Hartmann, 1821 (currently *Discus ruderatus*). For the non-insider it is very difficult to see that a new name was established here. It is necessary to understand precisely the content of the German text: “s. meine Tab. II. f. 11. Der vorigen ähnlich, aber aufgeblasener, rauher, weniger Umgänge“ (= see my plate II, figure 11. Similar to the previous one, but more inflated, rougher, fewer whorls).

Sherborn had to analyse difficult and chaotically arranged works, works for which extracting one new name took much longer than in carefully compiled works. Difficult works became increasingly common after 1790. One example is Hartmann (1821), written in German in Fraktur script with 64 new names of molluscs, of which 6 are used today (Fig. 3). New names in this work were not indicated to be new, so it was very difficult to see in the work which names were new and which ones had previously been established. In such a case it is necessary to check every single mentioned name, to see if it had previously been established or not. Without a computer this is a time-consuming process.

Of those that were new, it was difficult to see if they had a description, because no Latin diagnoses were given and many different topics were discussed in the text. Some names were subsequently used and placed into a different genus, so that it requires either additional research to find such previously established names in the database, or special malacological knowledge, or understanding Hartmann’s German Fraktur text in which he explained that the name was subsequently used from a previous work where it had been classified in a different genus.

Sherborn listed 24 names as new, only 17 of which were correct (two are used today), five were attributed to incorrect works (three are used today).

### Hartmann (1821): new names and names extracted by Sherborn

- 17 names were listed correctly (among these were *Acicula* Hartmann, 1821 and *Hydrobia* Hartmann, 1821)
- 5 species were missed: 2 new species were overlooked, 1 name with figure was marked as a nomen nudum, 2 names were incorrectly listed as available for previous works where they had been nomina nuda. Two of the five names of this fraction are used today: *Ciliella ciliata* (Hartmann, 1821), *Discus ruderatus* (Hartmann, 1821) (Fig. 3).
- 41 varieties were missed. Sherborn systematically ignored all names established for varieties. Two of these 41 names are used today for species: *Radix ampla* (Hartmann, 1821), *Trochulus clandestinus* (Hartmann, 1821).
- 1 species had an incorrectly cited genus (*Clausilia parvula* was incorrectly listed in *Auricella*). A specific name listed with an incorrect genus cannot be recognised in the list.
- 1 nomen nudum was listed as new (*Planorbis dubius*). This name had no description.
- 5 names were established in previous sources and subsequently used by Hartmann, but were listed as new for Hartmann by Sherborn. In three cases Hartmann gave bibliographical references which were not verified by Sherborn, in one case a German explanation was not understood. Three names are used today: *Acicula lineata* (Draparnaud, 1801), *Oligolimax annularis* (Studer, 1820), *Pomatias* Studer, 1789.

Incomplete research (not checking all cited sources, not verifying the presence of a true description in a foreign language) was probably a strategy to avoid spending too much time on difficult works with low numbers of new names. We know from Sherborn’s correspondence he felt a sense of continuous pressure and responsibility for his huge task (Evenhuis 2016, Shindler 2016) and he may have chosen to adopt a strategy of avoiding situations of greatly diminishing returns.

In the AnimalBase team we did not skip such difficult works, as our objective was to test the existing historical data. This had the effect that some highly-skilled team members had to spend a significant amount of time with extremely low output in terms of numbers of new taxonomic names extracted per time unit.

We were often surprised to observe that Sherborn did not always verify bibliographical references given in the original sources, to see if a name was established in a previous work and thus, not new in the reference in front of him. Similar issues are faced by authors of applications to the ICZN Commission, and are often caught by careful work by the editorial work on the *Bulletin of Zoological Nomenclature* (E. Michel 2013). Checking sources was perhaps easier for the AnimalBase team than it was for Sherborn, when a digitised book was often only one mouse-click away, and more time consuming for Sherborn who had to go to the shelf, or request loans from far away libraries, to get the printed book.

### The danger zones for high error rates when working with *Index Animalium*

How dangerous is it to rely on Sherborn’s work, and where do we have to pay special attention? This question cannot be answered easily or generally. Taxonomists work with Sherborn’s *Index Animalium* under various different objectives. The main objective of our team was to extract all available names from a published work. Doing this we had to be aware that Sherborn’s list was very slightly incomplete. We also knew that if no Latin diagnoses were given and works were in German, Swedish or French, we had to pay more attention. But this was probably an unusual form of working with the *Index*.

More commonly, taxonomists will look in *Index Animalium* for the original source of a name that was established before 1851. Here they will be confronted with the danger of not finding the name, or finding the name recorded from an incorrect source.

In 2–3% of the cases the name will not be found, and in some taxonomic groups more. Sherborn missed various important works with different impact in various groups. In those animal groups where many varieties were named before 1851 the danger of missing names is generally higher. As a general rule, Sherborn’s record provides higher reliability for insects than for molluscs, but also within insect taxa there seem to be differences. It is always necessary to ask colleagues working in the same area and with some experience in bibliographic work, for their judgement on the reliability on Sherborn’s *Index*.

Taxonomists working on fossil species will also know the extent to which they can rely on Sherborn, as this can vary significantly.

Finding the name recorded with an incorrect source will happen more often. This also differs by taxonomic group, for the same reason just explained above. If important standard works were missed, the names were attributed to incorrect subsequent sources. If non-binominal works were cited as original sources, such as Gronovius (1763, 1781), Meuschen (1778) or Martyn (1784), it is highly probable that the source will be incorrect. This is easy to see, however in the next step without Sherborn’s help it is very difficult to find the correct source in such a case.

Others might consult Sherborn to determine the orthography of a name’s original spelling. These researchers may run the danger of finding the name incorrectly spelled in the *Index*. This danger is generally very low, however in disputed names, for example in unusual Latin words, incorrect information may have accumulated in *Index Animalium*. We came to the general conclusion that if the spelling of a name has subsequently been the subject of debate, it is always necessary to consult the original source and not rely on the *Index* alone.

Again others might consult the *Index* to see if a name was actually made available in a certain work or not. They will run the risk of finding an unavailable name being incorrectly marked as available in the *Index*, or an available name recorded as unavailable. Insects such as aphids or moths with specific host plants are particularly problematic in this regard, as they were often presented without description in the original sources. In Sherborn’s *Index*, no indication is visible that these names were not made available in those sources. Generations of scientists have relied on Sherborn’s information and it comes often as a great surprise to see that a name of a well-known and frequent species established more than 200 years ago cannot be attributed to a certain work because no description was given. The moths in [Denis and Schiffermüller] (1775) are the best example. Hundreds of names of this extremely important work were incorrectly regarded as available for many decades. For many names their unavailability from that work and their correct original sources are yet to be discovered. Interestingly, also some Linnean names presented in his 1758 work with host plants have this problem.

Taxonomists should probably not consult Sherborn to verify the correct authorship for a name. For this task it is necessary to consult the original source and to apply the modern rules, especially if various different authorships were cited in various literature sources. As a general rule it could perhaps be said, if two or more authorships were given for a name in various different recent literature sources, the authorship cited by Sherborn would quite probably be the incorrect one.

## Final conclusions

The main conclusion we can derive for future projects aimed to extract complete lists of names from original sources is that if the size of the project passes a certain limit, then doing this without errors is effectively impossible. We would not recommend attempting production of a ‘perfect list’. This applies particularly to the idea of establishing Lists of Available Names (LANs, Code Art. 79). In lists of 2000 or 3000 names it might be possible with reasonable costs in terms of time and energy to have a list free of error in the spellings and the original sources of the names. If the number of names passes this barrier, the time needed to get the record complete will increase rapidly towards infinity.

Manual compilation of large nomenclators will always face an error rate of at least 2-4%, no matter how thoroughly they are researched and how many people contribute. If more people contribute, like the AnimalBase team, the increase in coverage is counterbalanced by the difficulty in maintaining a common standard, which potentially leads to higher error rates. If only one person contributes, like in Sherborn’s case, nobody is there for a control, but the experience shows that the record can be more reliable. If more data are to be recorded besides name and original source, such as in the ZooBank project, then the error rates per entry would increase accordingly. If any such projects are aimed to provide official data resources they will need regulations what to do in those cases when the data record contains errors.

Sherborn was confronted with many problems that we also had in our own work. This included the difficulty in maintaining a common standard over time. We came to the conclusion that anyone who intends to repeat Sherborn’s job will inevitably be fascinated and awed by what he achieved, and particularly by his low non-systematic error rates.
